# Inborn errors of immunity in Canadian First Nations and Nunavut Inuit Children: the tip of the iceberg

**DOI:** 10.1093/pch/pxae026

**Published:** 2024-06-13

**Authors:** Anne Pham-Huy, Luis Murguia-Favela, Tamar Rubin, Sneha Suresh, Nicola A M Wright, Beata Derfalvi, Roona Sinha, Jennifer Bowes, Geoffrey D E Cuvelier, the Clinical Immunology Network-Canada (CINC), Rae Brager, Andrea Fong, Eyal Grunebaum, Vy Kim, Elie Haddad, Hélène Decaluwe, Fabien Touzot, Reza Alizadehfar, Alison Haynes

**Affiliations:** Division of Infectious Diseases, Immunology and Allergy, Children’s Hospital of Eastern Ontario, University of Ottawa, Ottawa, Ontario; Section of Hematology/Immunology, Alberta Children’s Hospital, University of Calgary, Calgary, Alberta; Division of Pediatric Allergy and Immunology, Children’s Hospital of Winnipeg, Department of Pediatrics and Child Health, University of Manitoba, Winnipeg, Manitoba; Division of iHOPE, Stollery Children’s Hospital, University of Alberta, Edmonton, Alberta; Section of Hematology/Immunology, Alberta Children’s Hospital, University of Calgary, Calgary, Alberta; Division of Immunology, IWK Health Centre, Department of Pediatrics, Dalhousie University, Halifax, Nova Scotia; Jim Pattison Children’s Hospital, University of Saskatchewan, Saskatoon, Saskatchewan; Division of Infectious Diseases, Immunology and Allergy, Children’s Hospital of Eastern Ontario, CHEO Research Institute, Ottawa, Ontario; Departments of Pediatrics and Oncology, University of Calgary, Alberta Children's Hospital, Calgary, Alberta; McMaster Children’s Hospital, McMaster University; Regina General Hospital, University of Saskatchewan; Hospital for Sick Children, University of Toronto; Centre Hospitalier Universitaire de Ste-Justine; Montreal Children’s hospital; McGill University Health Centre, McGill University; Janeway Children’s Health and Rehabilitation Centre, Memorial University

**Keywords:** First Nations and Inuit population, Inborn errors of immunity, Newborn screening, Primary immunodeficiency, Severe combined immunodeficiency

## Abstract

**Objectives:**

Inborn errors of immunity (IEI) are a heterogeneous group of genetic diseases that impact normal immune development and function. Individual IEI are rare, but collectively, can represent an important health burden. Little is known about the types of IEI seen in Canadian First Nations (FN) and Inuit populations. We sought to understand the spectrum of serious IEI in FN and Nunavut Inuit children, as a starting point for improving the awareness of these conditions in the community and for health care workers.

**Methods:**

A questionnaire was distributed to participating Canadian pediatric tertiary-care centers. Providers were asked to report cases of confirmed or suspected severe immunodeficiencies seen in FN and Nunavut Inuit children.

**Results:**

From 2004 to 2022, IEI were reported in 63 FN and 21 Inuit children by 4 pediatric hospitals across 3 Canadian provinces. The majority of cases were immunodeficiencies affecting cellular and humoral immunity (62% of cases in FN and 57% in Inuit children). IKBKB deficiency, adenosine-deaminase severe combined immune deficiency (SCID), and chronic granulomatous disease were the most common IEI. A wide variety of other IEI was reported, many of which would not be detected by current newborn screening for SCID and for which live-attenuated vaccines would have been contraindicated.

**Conclusions:**

IEI occur in FN and Inuit children and may be underrecognized. Better understanding the prevalence of these conditions in specific communities could help inform public health policies including newborn screening and immunization programs and ultimately improve the health of FN and Inuit children in Canada.

Indigenous peoples living in what is now called Canada, are reported to have a disproportionate burden of health issues compared to non-Indigenous Canadians. This includes higher rates of lower respiratory tract infections (1–4), chronic medical conditions (5), perinatal mortality, and hospitalization (6,7). These increased incidences are often attributed to socioeconomic factors and living in remote settings (8). Clinical immunologists in Canada, however, have suspected through experience, that inborn errors of immunity (IEI) might also be involved. IEI, also known as primary immunodeficiencies, are a heterogeneous group of genetic diseases that impact immune development and function. The last two decades has seen an exponential increase in newly defined IEI, with over 400 distinct conditions now described under 10 categories (9). In general, IEI are rare. However, in small and geographically isolated populations with potential for founder pathogenic variants, the incidence may be much higher (10,11). A Canadian pediatric surveillance study of SCID, for instance, noted an incidence of 1:71,000 in Canadian non-Indigenous compared with 1:23,000 in Canadian Indigenous populations (12).

Since 2013, population-based newborn screening (NBS) programs for SCID have been implemented across most of Canada (Table 1). Detecting SCID by NBS promotes prompt diagnosis, early interventions, such as life-saving hematopoietic stem cell therapy (HSCT). All programs use the T cell receptor excision circle (TREC) assay as a biomarker for abnormal T cell development, with a low or undetectable TREC level being a positive screen for SCID. However, this assay does not detect all severe primary immunodeficiencies. For example, TREC levels are normal at birth for newborns with the inhibitor of nuclear factor kappa-B kinase subunit beta (IKBKB) deficiency, meaning the disorder is missed on NBS for SCID. A report by Pannicke et al. initially described four cases of IKBKB deficiency in a Northern Cree community in Manitoba that would have not been identified by standard SCID NBS (13). This led to a pilot project followed by implementation of an expanded NBS program in Manitoba for the early diagnosis of IKBKB deficiency using direct mutational analysis on dried blood spots (14). Infants detected with IKBKB deficiency are now isolated immediately after birth, with HSCT performed between 1 and 2 months of age, before the onset of life-threatening infections (15).

**Table 1.** Newborn screening for severe combined immunodeficiency (SCID) programs in Canada

Province/territory	Year of program implementation
Ontario	2013
Nova Scotia	2016
Prince Edward Island	2016
New Brunswick	2016
Alberta	2019
Northwest Territories	2019
Manitoba	2020
Nunavut	2020 (Kivalliq) 2016 (Kitikmeot) 2015 (Qikiqtaaluk)
Saskatchewan	2022
British Columbia	2022
Yukon	2022
Quebec	2023
Newfoundland and Labrador	No program

In many severe IEI, live-attenuated vaccines are contraindicated due to the risk of vaccine-associated disease. In Canada, routine live viral vaccines (LVV) include: the rotavirus, measles–mumps–rubella (MMR), and varicella vaccines. FN or Inuit children living in the Northwest Territories, Northern Manitoba, Northern Quebec, and Nunavut also receive (soon after birth) the Bacille–Calmette–Guérin (BCG) vaccine, a live bacterial vaccine, to prevent disseminated tuberculosis. Severe complications, including death from the BCG vaccine, have been reported in Indigenous children with unrecognized primary immunodeficiency (15–17). Failure to recognize an IEI may therefore result in an unintended fatality from administration of a live-attenuated vaccine. An example of how better understanding a primary immunodeficiency within a community can help inform public health policies is again illustrated with IKBKB deficiency in the two described Northern Cree communities of Manitoba (14). Prior to the screening program, BCG immunization was halted for all infants due to reported deaths. Since implementing the NBS program for IKBKB deficiency, BCG vaccination was safely re-implemented for infants screening negative for the variant. Knowing the specific genetic variant within a community led to a program that improved newborn diagnosis and early interventions.

The burden of IEI in Canadian FN and Inuit children is unknown. This project was aimed as a starting point to report the types of IEI seen in First Nations and Inuit children in three Canadian provinces and one territory as a means to improve understanding for specialists, frontline health care workers, and participating FN and Inuit communities. This information could aid in resource allocation and guide health policies such as newborn screening and vaccination.

## METHODS

### Study design

**Figure 1.**
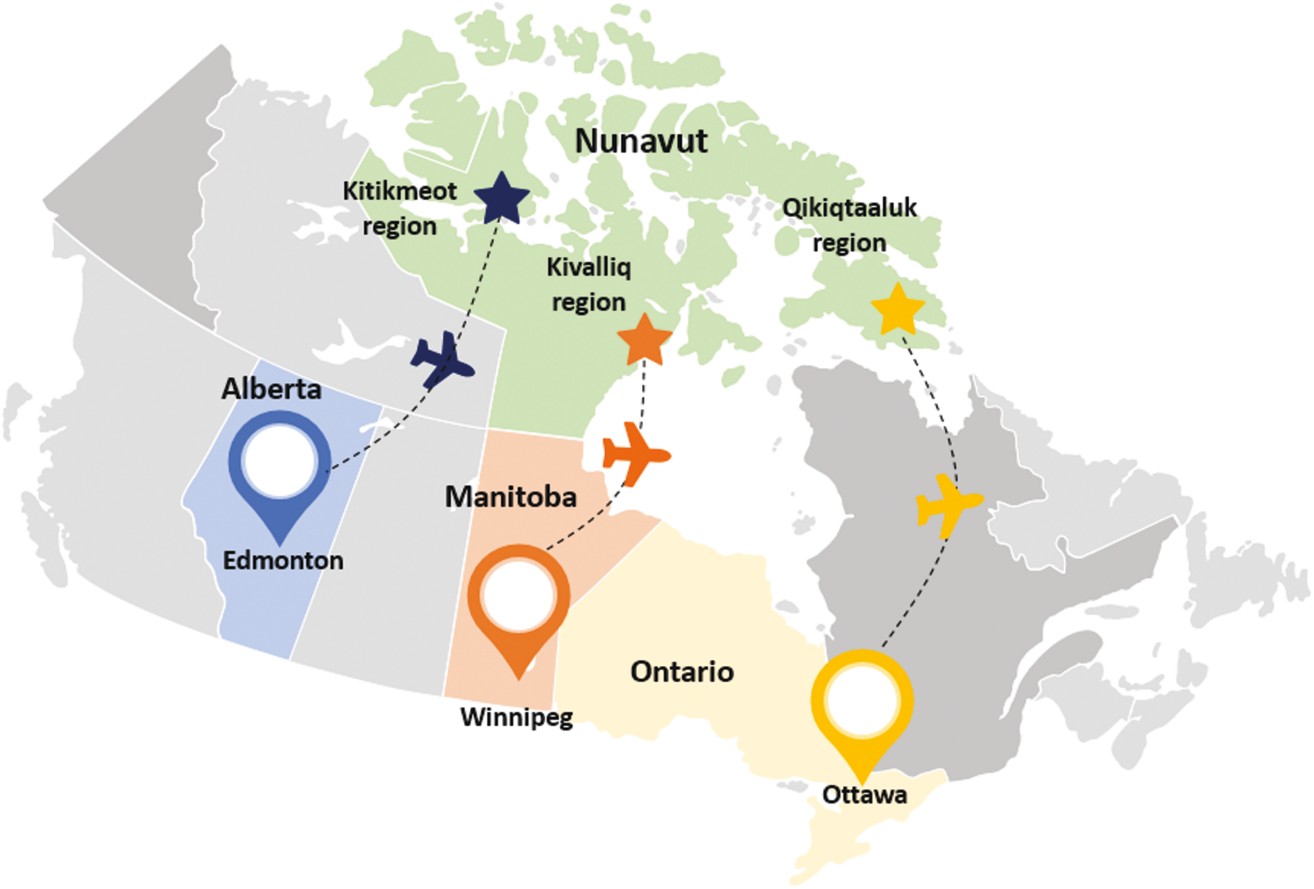
Catchment area and travel routes for Nunavut Inuit children needing pediatric tertiary-care health services. *Created using*www.presentationgo.com

Twelve Canadian tertiary-care pediatric centers that provide clinical immunology services were voluntarily invited to participate, of which four accepted (CHEO, Ottawa; Winnipeg Children’s Hospital, Winnipeg; Alberta Children’s Hospital, Calgary; and Stollery Children’s Hospital, Edmonton). A questionnaire was distributed to each center detailing the types of IEI managed through each program in FN and Inuit children. In Canada, specialized pediatric care for Nunavut Inuit children is provided in urban pediatric tertiary-care centers, with each center assigned a specific catchment area (Figure 1). It is expected that all children with severe presentations coming from Nunavut would be transferred for care and managed at these pediatric centers. The study period included patients seen between January 1, 2004, and September 20, 2022. Providers reviewed clinic registries and patient lists for cases. Investigators were asked to report cases of a FN or Inuit child diagnosed with severe forms of IEI according to IUIS categories (9). Patients were required to be identified as being of First Nations Peoples of Canada or Inuit of Nunavut, as reported in the clinical chart. We excluded Metis heritage due to the difficulty in acquiring this information with accuracy from databases and charts. We excluded immunodeficiencies presenting with typically milder courses such as selective IgA deficiency, isolated IgG subclass deficiencies, or partial DiGeorge syndrome. In order to protect the anonymity of the patient and the community, we did not collect data on specific location, but rather on the broad area (i.e., province or territory). We did not collect specific data on demographics, family history, outcome, vaccination, or therapy received. When a specific diagnosis or molecular diagnosis was unknown, the investigator was asked to provide a brief description of the phenotype and corresponding classification.

Once a case was identified, the main authors (APH, GC) classified each defect or clinical syndrome according to whether or not it would be expected to be detected by NBS using the TREC assay and if there was a contraindication to LVV, BCG vaccine, or both. In cases where the molecular diagnosis was unknown, we classified the case as being unknown. We also described the standard therapy used for the management of the specific IEI, including HSCT, immunoglobulin replacement therapy (IgRT), or other, in order to better understand the impact of early and prompt diagnosis. For cases that had an unknown genetic variant and was specific to the reported patient, management was described as being unknown.

### Ethics and support

This study was initiated by clinicians caring for Indigenous children with severe primary immunodeficiencies who expressed the need to better understand the types of conditions seen across various First Nations and Inuit communities. To ensure that this was also a perceived need for Indigenous communities, the study team invited various organizations such as the Assembly of First Nations (AFN) and Inuit Tapiriit Kanatami (ITK) for input and collaboration (Spring 2019). AFN referred our study team to the First Nations Information Governance Center (FNIGC) and ITK to the Nunavut Tunngavik Incorporated (NTI), who both provided input in the protocol development and study design (Fall 2019).

Study approval was granted by the Research Ethics Boards of the University of Ottawa, University of Calgary, University of Manitoba, and University of Alberta. In addition, FNIGC, NTI, the Alberta First Nations Information Governance Centre, the First Nations and Inuit Health Branch–Alberta region, and the Ministry of Health of Nunavut provided letters of support for this project.

Results of this study were presented to members of the FNIGC and NTI in March 2023. The draft manuscript was further circulated amongst this group for further input in May-June 2023.

## RESULTS

**Figure 2.**
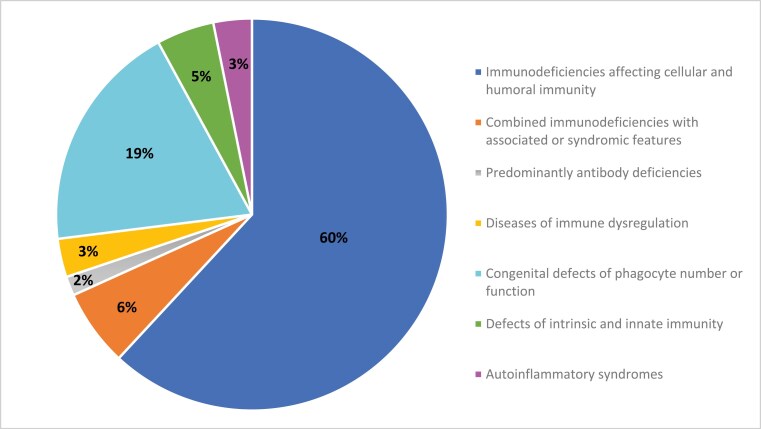
Proportion of inborn errors of immunity by classification in First Nations Children

**Figure 3.**
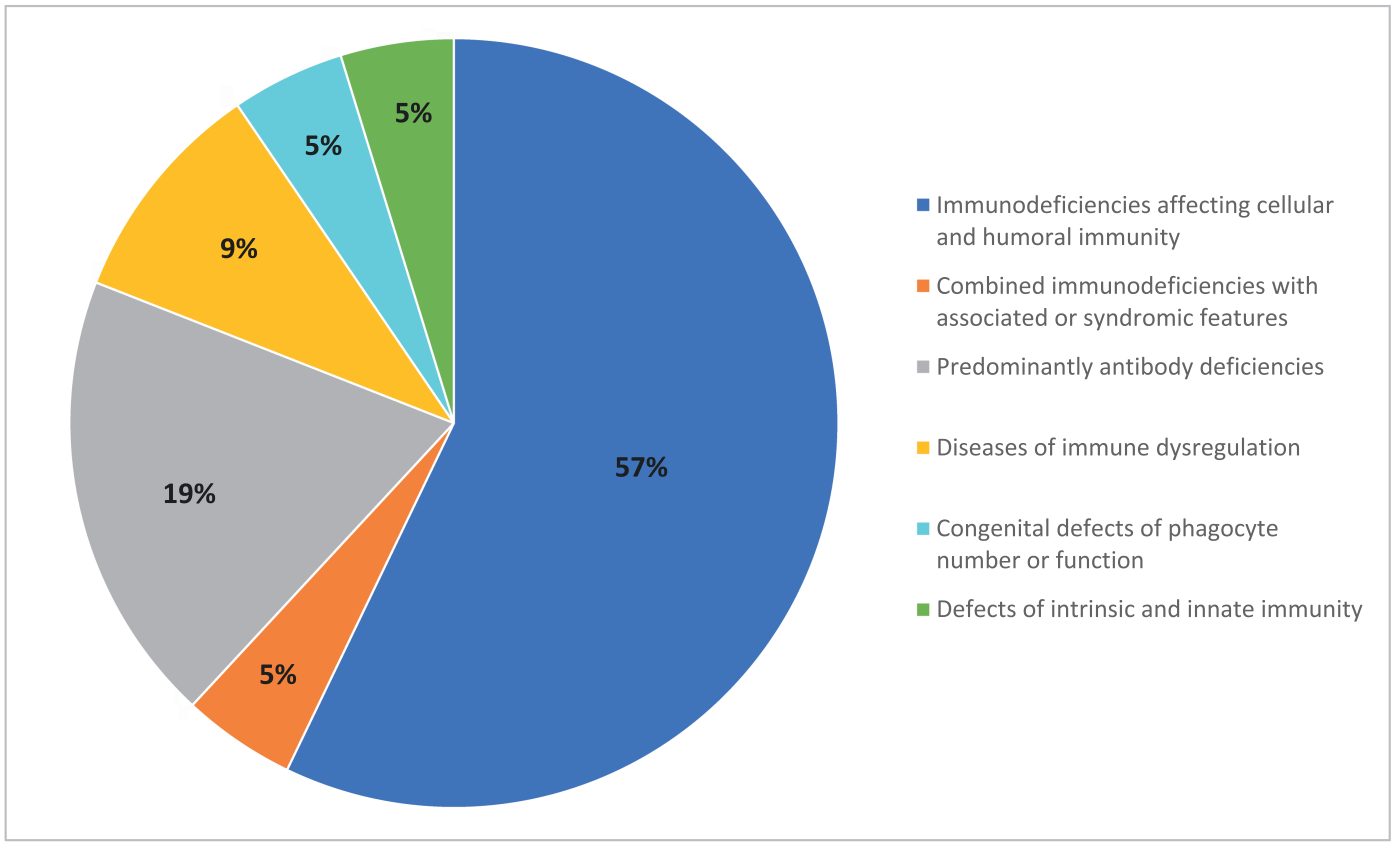
Proportion of inborn errors of immunity by classification in Nunavut Inuit Children

IEI were reported in 63 FN children and 21 Nunavut Inuit children (Figures 2 and 3; Tables 2 and 3). Of these, 42 (67%) FN and 14 (67%) Inuit children had confirmed molecular diagnoses. In both FN and Inuit children, combined immunodeficiencies were the most commonly reported (60% of cases in FN and 57% in Inuit children). Two cases of SCID were identified in FN infants and 10 in Inuit children. Importantly, seven cases of adenosine-deaminase (ADA) deficiency SCID were reported in Inuit infants from the Qikiqtaaluk region of Nunavut. Although we excluded cases of 22q11 microdeletion leading to “partial DiGeorge syndrome,” we did include cases of complete DiGeorge syndrome resulting in a SCID phenotype. One such case was reported in a First Nations infant. IKBKB was the most frequent single primary immunodeficiency reported in 18 FN children, mostly from Manitoba, but also from Saskatchewan and Alberta. Sixteen of these children have been previously reported (15). There was one case of MHC Class II deficiency, also called Bare lymphocyte syndrome type 2 (due to a homozygous RFX5 variant).

**Table 2.** Inborn errors of immunity identified in First Nations children

Inborn error of immunity	Number of cases reported	Province/territory of case	Standard therapy	Detected by TREC assay	Vaccine contraindicated	
					LVV	BCG
**Immunod** **eficiencies affecting cellular and hu** **moral immunity**						
SCID T-B + NK+ (unknown gene)	1	Alberta	HSCT	Likely	Yes	Yes
SCID—Omenn syndrome (unknown gene)	1	Alberta	HSCT	Likely	Yes	Yes
IKBKB deficiency (IKBKB variant)	18	Manitoba (14) Saskatchewan (3) Alberta (1)	HSCT	No	Yes	Yes
MHC Class II (RFX5 variant)	1	Alberta	HSCT	Possible	Yes	Yes
CARD11 deficiency (SCID phenotype)	2	Alberta	HSCT	No	Yes	Yes
Suspected CID: Severe MRSA infection	10	Manitoba	Unknown	Unknown	Yes	Yes
Suspected CID: Severe H influenza infection	4	Manitoba	Unknown	Unknown	Yes	Yes
Suspected CID: NOS	1	Alberta	Unknown	Unknown	Yes	Yes
**Co** **mbined immunodeficiencies with associated or syndromic features**						
22q11 microdeletion syndrome Complete Digeorge	1	Alberta	Thymic transplant	Yes	Yes	Yes
Ectodermal dysplasia with immunodeficiency (TRAF6 variant)	3	Alberta	IgRT HSCT	No	Yes	Yes
Ectodermal dysplasia with immunodeficiency (unknown gene)	1	Manitoba	IgRT	No	Yes	Yes
**Predomina** **ntly antibody deficiencies**						
Common variable immunodeficiency	1	Manitoba	IgRT	No	Caution	Caution
**Diseas** **es of immune dysregulation**						
SAP Deficiency (X-linked lymphoproliferative disorder type 1) (SH2D1A variant)	1	Alberta	Unknown	No	Yes	Yes
Familial HLH 3 Munc 13-4 deficiency (UNC13D variant)	1	Alberta	HSCT	No	Yes	Yes
**Congenital defe** **cts of phagocyte number or function**						
Chronic granulomatous disease (NCF2 variant)	3	Saskatchewan	HSCT	No	No	Yes
X-linked chronic granulomatous disease (CYBB variant)	7	Manitoba (4) Alberta (3)	HSCT	No	No	Yes
GATA2 deficiency	1	Alberta	Unknown	No	Yes	Yes
Phagocytic defect NOS	1	Manitoba	Unknown	No	No	Yes
**Defects in intri** **nsic and innate immunity**						
ZNFX1 deficiency	1	Manitoba	Unknown	No	Yes	Caution
IRF8 deficiency	1	Alberta	Unknown	No	Yes	Caution
Mendelian susceptibility to mycobacterial disease (MSMD) Unknown variant	1	Manitoba	Unknown	No	No	Yes
**Autoinflammato** **ry syndromes**						
NCKAP1L (or HEM1) deficiency (NCKAP1L variant)	2	Manitoba	Unknown	No	Yes	Caution
Total patients reported	63 cases					

*HLH Hemophagocytic lymphohistiocytosis; HSCT Hematopoietic stem cell transplantation; IgRT Immunoglobulin replacement therapy; NOS Not otherwise specified; LVV Live viral vaccine; BCG Bacille–Calmette–Guerin*

**Table 3.** Inborn errors of immunity identified in Nunavut Inuit children

Inborn error of immunity	Number of cases reported	Nunavut region	Standard therapy	Detected by TREC assay	Vaccine contraindicated	
					LVV	BCG
**Immunodeficiencies affecting cellular an** **d humoral immunity**						
SCID T-B-NK- (ADA deficiency)	7	Qikiqtaaluk	HSCT	Yes	Yes	Yes
SCID T-B+ (CORO1A variant)	1	Qikiqtaaluk	HSCT	Yes	Yes	Yes
SCID T-B-Artemis (DCLRE1C variant)	1	Kivalliq	HSCT	Yes	Yes	Yes
Suspected CID NOS	2	Kivalliq	HSCT	Unknown	Yes	Yes
**Combined immunodeficiencies with** **associated or syndromic features**						
Suspected CID Developmental delay, congenital abnormalities and fulminant infections	1	Kivalliq	Unknown	Unknown	Yes	Yes
**Predominantly antib** **ody deficiencies**						
PIK3CD (GOF)	1	Kitikmeot	Unknown	No	No	No
Specific antibody deficiency	3	Kivalliq (2) Qikiqtaaluk (1)	Antibiotic prophylaxis	No	No	No
**Diseases of immune** **dysregulation**						
RIPK1 deficiency	1	Kitikmeot	Unknown	No	No	No
HLH (unknown gene)	1	Kivalliq	Unknown	No	Yes	Yes
**Congenital defects of phagocyte** **number or function**						
X-linked chronic granulomatous disease (CYBB gene)	1	Qikiqtaaluk	HSCT	No	No	Yes
**Defects in intrinsic** **and innate immunity**						
IFNAR2 deficiency	1	Qikiqtaaluk	IRT	No	Yes	Yes
Total patients reported	21 cases					

*HLH Hemophagocytic lymphohistiocytosis; HSCT Hematopoietic stem cell transplantation; IRT Immunoglobulin replacement therapy; RIPK1: Receptor-interacting serine/threonine-protein kinase 1; NOS Not otherwise specified; LVV Live viral vaccine; BCG Bacille–Calmette–Guerin*

Ten cases of CGD were identified in FN children and 1 case in an Inuk child. Another child of FN descent was identified to have IRF8 deficiency presenting with a phenotype compatible with a Mendelian susceptibility for mycobacterial disease (MSMD). A novel pathogenic variant in NCKAP1L gene (also known as HEM1 variant) leading to NCKAP1L deficiency was reported in two FN children. One of these cases was previously reported in a FN child presented with recurrent infections, hepatosplenomegaly, bronchiectasis, and antibody abnormalities (18). Many sites reported cases of children presenting with severe infections (due to viruses, methicillin-resistant *Staphylococcus aureus*, *Hemophilus influenzae*) but without a molecular diagnosis.

Given the severity of the IEI reported, the majority would have had a contraindication to receiving LVV or BCG vaccine. Of the cases identified, 50/63 (79.3%) of FN children with identified IEI should not receive any LVV and 58/63 (92%) should not receive the BCG vaccine. For Nunavut Inuit children, 15/21 (71.4%) of those found to have an IEI should not receive any LVV and 16/21 (76.2%) should not receive the BCG vaccine.

## DISCUSSION

Frontline healthcare workers, including nurses, family physicians, and pediatricians working with FN and Inuit populations, frequently encounter infants and children with a variety of infectious diagnoses. The possibility of an underlying IEI should be considered, particularly when infections are recurrent, severe, or associated with the presence of autoimmunity, hyperinflammation, and immune dysregulation. Using contemporary diagnostic criteria, we show that a variety of IEI are documented in FN and Nunavut Inuit populations of Canada. Unfortunately, our study, not being population based, is unable to estimate the true incidence of IEI in FN and Inuit children. As such, we believe our data may be only the “tip of the iceberg” regarding the types and burden of IEI in FN and Inuit children. Nonetheless, this data is important as a starting point to increase recognition of IEI in FN and Inuit children among frontline healthcare workers and FN and Inuit communities, so that diagnosis can be expedited, and public health policies such as LVV and BCG vaccination administration amended and improved. Importantly, basic immune work-up such as a complete blood count or serum immunoglobulins may be normal in many IEI. A high index of suspicion is warranted and prompt referral to pediatric immunology is important for suspected cases. Furthermore, providers should also be aware that current NBS programs, while excellent for detecting SCID, will miss a number of severe IEI that could be present in select populations.

One of the more frequently reported immunodeficiencies in FN children in our survey was IKBKB combined immunodeficiency. These patients originated from two small FN communities in Northern Manitoba, where over decades, providers treated young Northern Cree infants presenting with severe and life-threatening infections, including fatal disseminated *Mycobacterium bovis* following BCG vaccination (15). Before knowledge of the IKBKB variant, BCG vaccination was discontinued for all infants in these two communities from these observations. In 2013, homozygous variants in the IKBKB gene (c.129dupG), leading to a complete deficiency of IKKβ expression was described in individuals from these two communities (13). Unlike classical SCID which has T cell lymphopenia, IKBKB deficiency results in normal to elevated levels of T and B cells that are nonfunctional and therefore can be missed on standard NBS for SCID due to normal TREC levels. This variant is a classic founder mutation seen in geographically isolated populations. Carriage rate state studies have reported an estimated incidence of a newborn carrying IKBKB variants (therefore being affected) to be 1 in 686 births (14). Over subsequent years, cases were diagnosed in Saskatchewan and Alberta as people moved, and our suspicion is that additional cases in Western Canada were likely never diagnosed in FN infants who died of fulminant infections.

We identified nine cases of SCID in Inuit children, with seven due to ADA deficiency. All were from the Qikiqtaaluk region of Nunavut and reported in the last decade. ADA deficiency accounts for 10–15% of SCID cases, with a reported incidence of approximately 1 in 200,000–500,000 live births (19,20). The reported number of births per year for Nunavut in 2020 was 839 (21), suggesting a significantly higher incidence of ADA deficiency in this region, roughly estimated in the range of 1: 600 to 1:2000. Without appropriate diagnosis, ADA-SCID is a fatal disease. Fortunately, ADA deficiency is detected by SCID NBS. If treated early with HSCT or gene therapy, before the onset of active infections, overall survival in ADA-SCID is reported to be 91% and 100%, respectively (22).

Eleven cases of chronic granulomatous diseases (CGD) were reported (n = 10 FN and n = 1 Inuit). CGD is a phagocytic defect leading to increased susceptibility to bacterial and fungal infections. BCG vaccine is contraindicated in these patients as disseminated and fatal disease can occur (17). Standard of care for these children is antimicrobial prophylaxis and early HSCT. Human IFNAR2 deficiency is an innate immunodeficiency impacting type 1 interferon antiviral response. It was previously described in a 13-month-old infant diagnosed with fatal measles encephalitis following administration of the MMR vaccine (23). Further cases describe severe viral infections, complications of LVV, or hemophagocytic lymphohistiocytosis (HLH). A novel missense p.Ser53Pro variant in IFNAR2 has been associated with Inuit descent (24,25) In our study, we report one Inuk child with this variant. These cases highlight the importance of this immune pathway and the catastrophic consequences of administrating a live vaccine to an unrecognized immunodeficiency. We also identified several other IEI as single cases (Tables 2 and 3), many of which are extremely rare (e.g., ZNFX1, IRF8, CARD11, NCKAP1L, CORO1A). Importantly, none of these IEI are currently picked up by NBS using the TREC assay.

## CONCLUSION

A comprehensive picture of all IEI in FN and Inuit children in Canada remains unknown. Challenges remain in conducting a study on a national level. A national IEI patient registry would be instrumental to better identify rare immunodeficiencies with specific gene variants enriched in certain geographic or ethnic regions. Nonetheless, we feel this data is important to improve awareness of IEI in FN and Inuit children of Canada, promote universal access to SCID NBS, and begin to evaluate public health policies such as vaccination practices. We show that multiple different IEI are seen in FN and Inuit children at four pediatric tertiary-care centers in Canada, leading to a wide variety of clinical syndromes. Our study spans nearly two decades, from 2004 to 2022, corresponding to advances in immunologic and genetic testing that allow for more precise IEI diagnosis. Our data suggest that IEI may be underrecognized in Indigenous populations and could explain some of the higher burden and severity of infectious diseases seen in certain communities.

**Members of the Clinical Immunology Network-Canada (CINC)**

Rae Brager, MD, McMaster Children’s Hospital, McMaster University, Andrea Fong, MD, Regina General Hospital, University of Saskatchewan, Eyal Grunebaum, MD, Vy Kim, MD, Hospital for Sick Children, University of Toronto, Elie Haddad, MD, Hélène Decaluwe, MD, Fabien Touzot, MD, Centre Hospitalier Universitaire de Ste-Justine, Reza Alizadehfar, MD Montreal Children’s hospital, McGill University Health Centre, McGill University, Alison Haynes, MD, Janeway Children’s Health and Rehabilitation Centre, Memorial University.
